# A simple method for estimating time-irreversible nucleotide substitution rates in the SARS-CoV-2 genome

**DOI:** 10.1093/nargab/lqae009

**Published:** 2024-02-02

**Authors:** Kazuharu Misawa, Ryo Ootsuki

**Affiliations:** Department of Human Genetics, Yokohama City University Graduate School of Medicine, 3-9 Fukuura, Kanazawa-ku, Yokohama 236-0004, Japan; RIKEN Center for Advanced Intelligence Project, 1-4-1 Nihonbashi, Chuo-ku, Tokyo 103-0027, Japan; Department of Natural Sciences, Faculty of Arts and Sciences, 1-23-1 Komazawa, Setagaya-ku, Tokyo 154-8525, Japan; Department of Chemical and Biological Sciences, Faculty of Science, Japan Women's University, 2-8-1 Mejirodai, Bunkyo-ku, Tokyo 112-8681, Japan

## Abstract

SARS-CoV-2 is the cause of the current worldwide pandemic of severe acute respiratory syndrome. The change of nucleotide composition of the SARS-CoV-2 genome is crucial for understanding the spread and transmission dynamics of the virus because viral nucleotide sequences are essential in identifying viral strains. Recent studies have shown that cytosine (C) to uracil (U) substitutions are overrepresented in SARS-CoV-2 genome sequences. These asymmetric substitutions between C and U indicate that traditional time-reversible substitution models cannot be applied to the evolution of SARS-CoV-2 sequences. Thus, we develop a new time-irreversible model of nucleotide substitutions to estimate the substitution rates in SARS-CoV-2 genomes. We investigated the number of nucleotide substitutions among the 7862 genomic sequences of SARS-CoV-2 registered in the Global Initiative on Sharing All Influenza Data (GISAID) that have been sampled from all over the world. Using the new method, the substitution rates in SARS-CoV-2 genomes were estimated. The C-to-U substitution rates of SARS-CoV-2 were estimated to be 1.95 × 10^−3^ ± 4.88 × 10^−4^ per site per year, compared with 1.48 × 10^−4^ ± 7.42 × 10^−5^ per site per year for all other types of substitutions.

## Introduction

Severe acute respiratory syndrome coronavirus-2 (SARS-CoV-2) is an RNA virus that has spread globally and is the cause of the current COVID-19 pandemic (1,2). The study of the molecular evolution of SARS-CoV-2 is important as it provides a better understanding of the dynamics of virus spread and transmission. Understanding molecular evolution is essential in developing effective vaccines, therapeutic approaches, and identification of viral strains. In addition, continuous surveillance of the evolution of the virus will contribute to the implementation of surveillance strategies and long-term preparedness against the disease. The main objective of this study is to propose a method for predicting nucleotide changes in SARS-CoV-2 genomes.

Genomic analyses of SARS-CoV-2 have demonstrated that 50% of the sequence mutations are cytosine-to-uracil (C-to-U) transitions with an 8-fold base frequency directional asymmetry between C-to-U and U-to-C substitutions (3–6). The asymmetric substitutions between C and U indicate that traditional time-reversible substitution models cannot be applied to the evolution of SARS-CoV-2 sequences, although many time-reversible nucleotide substitution models are available, including the Jukes-Cantor model (7), Kimura 2-parameter model (8), Hasegawa-Kishino-Yano model (9), Tamura and Nei model (10) and General Time-Reversible model (11). The molecular evolution of SARS-CoV-2 can be studied more effectively using time-irreversible models (12,13) than using time-reversible models. Previous studies of time-irreversible models have used iterative approaches, such as Newton-Raphson method, for estimating the substitution rates. Iterative methods require time-consuming calculations due to the repetition until estimates converge.

Here, we present a new time-irreversible model of nucleotide substitutions to estimate the substitution rates in SARS-CoV-2 genomes. In this study, we present a simple algorithm for estimating the substitution rates by using the diagonalization method. The diagonalization method is often used for time-reversible model, such as Hasegawa–Kishino–Yano model (9) and general time reversible model (11). To verify the new model, the number of nucleotide substitutions of genomic sequences of SARS-CoV-2 registered are investigated in this study. Genomic sequences from the Global Initiative on Sharing All Influenza Data (GISAID) (14) that have been sampled from all over the world were investigated in this study.

## Materials and methods

### Definition of substitution rate matrix

In this study, the process of nucleotide substitution is considered as a continuous Markov process. The four RNA bases C, U, G and A are designated as 1, 2, 3 and 4, respectively, and $p( {t,\ i,\ j} )$ is the probability that nucleotide $i$ is substituted by $j$ in time period $t$. ${{\bf P}}( t )$ is a matrix in which the *ij*th element is $p( {t,\ i,\ j} )$.

${{\bf P}}( t )$ satisfies the following Chapman–Kolmogorov equation:


(1) \begin{equation*}{{\bf P}}\left( {t + \Delta t} \right)\ = \ {{\bf P}}\left( t \right){{\bf P}}\left( {\Delta t} \right)\end{equation*}

Thus, equation (1) is obtained as


(2) \begin{eqnarray*}{{\bf P}}{\mathrm{^{\prime}}}\left( t \right) &= {\mathrm{\ }}\mathop {\lim }\limits_{{\mathrm{\Delta }}t \to 0} \frac{{{{\bf P}}\left( {t + {\mathrm{\Delta }}t} \right) - {{\bf P}}\left( t \right)}}{{{\mathrm{\Delta }}t}} = {{\bf P}}\left( t \right)\mathop {\lim }\limits_{{\mathrm{\Delta }}t \to 0} \frac{{{{\bf P}}\left( {{\mathrm{\Delta }}t} \right) - {{\bf P}}\left( 0 \right)}}{{{\mathrm{\Delta }}t}}\nonumber\\ & = {{\bf P}}\left( t \right){{\bf P}}^{\prime}\left( 0 \right)\end{eqnarray*}

### Time-irreversible model

Here, a new time-irreversible model is proposed for SARS-CoV-2 evolution. To model the directional asymmetry between C-to-U and U-to-C substitutions, a matrix, ${{\bf R}}$, was created, where $h$ is the C-to-U substitution rate and $a$ is the rate of other types of nucleotide substitutions.


(3) \begin{equation*}{{\bf R}} = \begin{pmatrix} - 2a - h & \quad h & \quad a & \quad a \\ a & \quad -3a & \quad a & \quad a \\ a & \quad a & \quad -3a & \quad a \\ a & \quad a & \quad a & \quad -3a \end{pmatrix} \end{equation*}

${{\bf R}}$ is a derivative of the substitution probability matrix with respect to time $t$ (3).

### Computing the powers of the substitution rate matrix by diagonalization

The substitution rate matrix ${{\bf R}}$ defined by equation (3) can be diagonalized as


(4) \begin{equation*}{{\bf S}} = {{\bf QR}}{{{\bf Q}}}^{- 1} = \begin{pmatrix} 0 & \quad 0 & \quad 0 & \quad 0 \\ 0 & \quad -b & \quad 0 & \quad 0 \\ 0 & \quad 0 & \quad -4a & \quad 0 \\ 0 & \quad 0 & \quad 0 & \quad -4a \end{pmatrix},\end{equation*}

where


(5) \begin{eqnarray*} && {{\bf Q}} = \begin{pmatrix} 4a & \quad -4a + 2b & \quad b & \quad b \\ 1 & \quad -1 & \quad 0 & \quad 0 \\ 0 & \quad 1 & \quad 0 & \quad -1 \\ 0 & \quad 0 & \quad 0 & \quad -1 \end{pmatrix}\nonumber\\ && {{\bf Q}}^{- 1} = \frac{1}{{4b}} \begin{pmatrix} 1 & \quad -4a + 4b & \quad 2b & \quad -b \\ 1 & \quad -4a & \quad 2b & \quad -b \\ 1 & \quad -4a & \quad -2b & \quad 3b \\ 1 & \quad -4a & \quad -2b & \quad -b \end{pmatrix},\nonumber\\ && *{5pc} {{\mathrm{and\ }}b = 3a + h.}\end{eqnarray*}

From equation (4), ${{\bf R}}$ is obtained as


(6) \begin{equation*}{{\bf R}} = {{{\bf Q}}}^{ - 1}{{\bf SQ}}\end{equation*}

A probability matrix, ${{\bf P}}( t )$, is obtained by


(7) \begin{equation*}{{\bf P}}\left( t \right) = \exp \left[ {t{{\bf R}}} \right] = \mathop \sum \limits_{k = 0}^\infty \frac{{{t}^k}}{{k!}}{{{\bf R}}}^k\end{equation*}

Thus,


\begin{equation*}{{\bf P^{\prime}}}\left( t \right) = {{\bf P}}\left( t \right){{\bf R}}\end{equation*}

Using equation (7), ${{\bf P}}( t )$ can be obtained by


(8) \begin{equation*}{{\bf P}}\left( t \right) = \exp \left[ {t{{\bf R}}} \right] = \mathop \sum \limits_{k = 0}^\infty \frac{{{t}^k}}{{k!}}{{{\bf R}}}^k = {{{\bf Q}}}^{ - 1}{{\bf T}}\left( t \right){{\bf Q}},\end{equation*}

where ${{\bf T}}( t )$ is defined by


(9) \begin{eqnarray*} {{\bf T}}\left( t \right) &=& \mathop \sum \limits_{k = 0}^\infty \frac{{{t}^k}}{{k!}}{{{\bf S}}}^k = \nonumber\\ && \begin{pmatrix} 1 & \quad 0 & \quad 0 & \quad 0 \\ 0 & \quad {\rm exp}[-bt] & \quad 0 & \quad 0 \\ 0 & \quad 0 & \quad {\rm exp}[-4at] & \quad 0 \\ 0 & \quad 0 & \quad 0 & \quad {\rm exp}[-4at] \end{pmatrix} \end{eqnarray*}

Finally, ${{\bf P}}( t )$ can be calculated by


(10) \begin{equation*}{{\bf P}}\left( t \right) = \begin{pmatrix} 1 - x -2z & \quad x & \quad z & \quad z \\ w & \quad 1 - w -2z & \quad z & \quad z \\ w & \quad y & \quad 1 - w - y - z & \quad z \\ w & \quad y & \quad z & \quad 1 - w - y - z \end{pmatrix}\nonumber\\ \end{equation*}

where


(11) \begin{equation*}\begin{array}{@{}*{1}{c}@{}} {c{\mathrm{\ }} = {\mathrm{\ }}a/b}\\ {w = c( {1 - {\mathrm{exp}}[ { - bt}]} )}\\ {x = ( {1 - c})( {1 - {\mathrm{exp}}[ { - bt} ]} ) - ( {1 - {\mathrm{exp}}[ { - 4at} ]} )/2}\\ {y = 1 - c( {1 - {\mathrm{exp}}[ { - bt} ]} ) - ( {1 + {\mathrm{exp}}[ { - 4at} ]})/2}\\ {z = ( {1 - \exp [ { - 4at} ]} )/4} \end{array}\end{equation*}

### Estimation of nucleotide substitution rates

Notably,


(12) \begin{equation*}x - y = \exp [ { - 4at}] - \exp [ { - bt}]\end{equation*}

where $at$ and $bt$ can be estimated using equation (6) and by solving simultaneous equations (11) and (12). The arithmetic mean is used when multiple estimates are obtained.

#### Estimation of nucleotide contents with respect to time

$N( {t,\ i,\ j} )$ is the observed number of cases where the ancestral nucleotide is $i$ and the derived nucleotide is $j$ in time $t$. ${{\bf N}}( t )$ is a matrix in which the *ij*th element is $N( {t,\ i,\ j} )$. The expected value of ${{\bf N}}( t )$ can be obtained by


(13) \begin{equation*}E\left[ {{{\bf N}}\left( t \right)} \right]\ = \ {{\bf N}}\left( 0 \right){{\bf P}}\left( t \right),\end{equation*}

where


(14) \begin{equation*}{{\bf N}}\left( 0 \right) = \begin{pmatrix} n(1) & \quad 0 & \quad 0 & \quad 0 \\ 0 & \quad n(2) & \quad 0 & \quad 0 \\ 0 & \quad 0 & \quad 2(3) & \quad 0 \\ 0 & \quad 0 & \quad 0 & \quad n(4) \end{pmatrix} ,\end{equation*}

and $n( i )$ is the number of nucleotides $i$ in the ancestral sequence.

If ${{\bf \hat{P}}}( t )$ is the estimate of ${{\bf P}}( t )$, ${{\bf \hat{P}}}( t )$ can be estimated by


(15) \begin{eqnarray*} && {{{\bf \hat{P}}}\left( t \right) = {{\bf N}}{{\left( 0 \right)}}^{ - 1}{{\bf N}}\left( t \right)}= \nonumber\\ && \begin{pmatrix} 1-x_{1}-z_{1} - z_{4} & \quad x_{1} & \quad z_{1} & \quad z_{4} \\ w_{1} & \quad 1 - w - z_{2} - z_{5} & \quad z_{2} & \quad z_{5} \\ w_{2} & \quad y_{1} & \quad 1-w_{2}-y_{1}-z_{6} & \quad z_{6} \\ w_{3} & \quad y_{2} & \quad z_{3} & \quad 1-w_{3} - y_{2} - z_{3} \end{pmatrix}\nonumber\\ \end{eqnarray*}

Using equation (16) we obtain estimated values for *w*, *x*, *y* and *z*.


(16) \begin{eqnarray*}\hat{w} &=& \frac{{{w}_1 + {w}_2 + {w}_3}}{3},\quad \hat{x} = {x}_1, \quad \hat{y} = \frac{{{y}_1 + {y}_2}}{2},\nonumber\\ && \hat{z} = \frac{{{z}_1 + {z}_2 + {z}_3 + {z}_4 + {z}_5 + {z}_6}}{6}\end{eqnarray*}

Applying equation (17) yields the estimated value for a and b.


(17) \begin{equation*}\hat{a} = - \frac{1}{4}\ln \left[ {1 - 4\hat{z}} \right],\quad \hat{b} = - \ln \left[ {1 - \hat{x} + \hat{y} - 4\hat{z}} \right]\end{equation*}

Equation (18) provides the estimated value for *h*.


(18) \begin{equation*}\hat{h} = \hat{a} - 3\hat{b}\end{equation*}

#### Confidence intervals of the estimates of the evolutionary rates

To obtain confidence intervals of the estimates of mutation rates, we used the bootstrap method. We perform bootstrap sampling by repeatedly creating new sets of the virus sequences of the same sample size through resampling with replacement from the original set of the sequences. This results in obtaining bootstrap samples. We created a distribution of the statistics obtained from the bootstrap samples. By examining the range from the 0.5th to the 99.5th percentile of the distribution, we obtained a 99% confidence interval.

### Sequence analysis of SARS-Cov-2

To verify the proposed model, the number of nucleotide substitutions of genomic sequences and the changes in nucleotide contents of SARS-CoV-2 were investigated. Genomic sequences of SARS-CoV-2 were retrieved from the GISAID database every six months from 31 December 2019 to 31 December 2021 (14). Samples used in this study were collected every six months. Gapped sites were excluded from the analysis. The sequence of the sample taken on 31 December 2019 was assumed to be the ancestral sequence, because it is the sequence first identified in Wuhan, China (gisaid_epi_isl: EPI_ISL_402125).

Genomes with >29 000 nucleotides were considered as having complete coverage. Sequences with <0.05% unique amino acid substitutions (i.e. substitutions not seen in other sequences in the database) and no insertions/deletions, unless verified by the submitter, were included in the analysis. Only sequences without undetermined (Ns) were used. A pairwise alignment of each genome sequence and the reference sequence was obtained using the MAFFT (15), which is a rapid tool for multiple sequence alignment. The substitution rates were estimated by comparing the sample sequences collected as of 31 December 2020 with the reference sequence. Given that 1792 sequences were collected on that date (as shown in Table 1), estimates were derived from these individual sample comparisons. We calculated the mean value of the estimates to determine the overall estimate and its standard error. The confidence intervals of the overall estimates were determined using the estimates of substitution rates obtained from pairwise comparisons. The date, region, and sample size details of the GISAID sequences used in this study are given in Table 1. To avoid sampling bias, we investigated the nucleotide changes in each region independently. Table 1 shows the sample size of each region.

**Table 1.** Date, region and sample size of the GISAID sequences used in this study

	Sampling date					
Region	2019/12/31	2020/6/30	2020/12/31	2021/6/30	2021/12/31	Total
Africa	0	19	11	13	5	48
Asia	1	70	211	334	440	1056
Europe	0	120	887	2369	1741	5117
North America	0	298	650	1057	558	2563
Oceania	0	52	13	11	28	104
South America	0	25	20	186	32	263
Total	1	584	1792	3970	2810	9157

### Synonymous and nonsynonymous changes of SARS-Cov-2 genes

To test whether the trend of nucleotide contents in the SARS-CoV-2 genome is caused by mutational bias or natural selection. the number of nonsynonymous and synonymous substitutions per site of the SARS-CoV-2 genes were estimated, because the selective force will depend on the function of the protein, which in turn depends on the amino acid sequences. Table 3 shows the number of nonsynonymous and synonymous changes per site of the SARS-CoV-2 genes estimated by NG86 model (16).

## Results

### Estimates of nucleotide substitutions of the SARC-Cov-2 genome

Table 2 shows the estimated substitution rates. C-to-U substitution rates were estimated as $1.95 \times {10}^{ - 3} \pm 4.88 \times {10}^{ - 4}$ per site per year, and for other types of substitutions the rates were $1.48 \times {10}^{ - 4} \pm 7.42 \times {10}^{ - 5}$ per site per year.

**Table 2.** Estimated substitution rate per site per year

Type	Rate	SD
Non C-to-U	1.48 × 10^−4^	7.42 × 10^−5^
C-to-U	1.95 × 10^−3^	4.88 × 10^−4^

**Table 3.** Major strains of SARS-CoV-2

Strain	Pango lineage
Reference	
Alpha	B.1.1.7
Beta	B.1.1.351
Gamma	P.2
S	A.23.1
Omicron	BA.1

### Changes in the nucleotide contents of the SARS-Cov-2 genome

**Figure 1.**
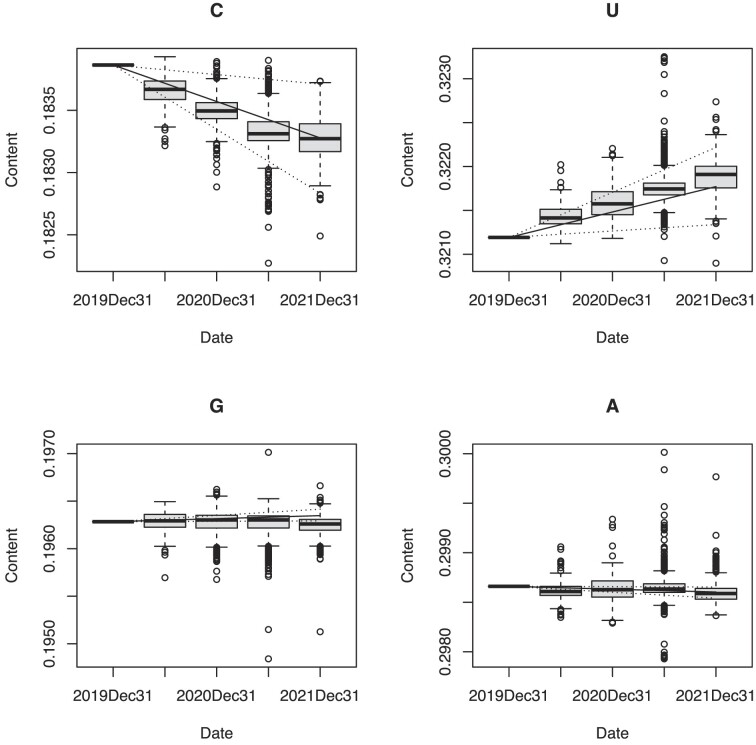
Bar plots of the changes in C (cytosine), U (urasil), G (guanine), and A (adenine) contents of the SARS-CoV-2 genomes and sample dates over the time period from 31 December 2019 to 31 December 2021, x axis: Date, y axis: Content. Solid lines represent trend curves depicting changes in nucleotide contents predicted by the new time-irreversible model. Dotted curves represent the 99% confidence intervals of the predicted nucleotide contents.

Bar plots of the changes in C content of the SARS-CoV-2 genomes and sample dates are shown in Figure 1. The results show that the number of Cs decreased in the SARS-CoV-2 genome over the time period from 31 December 2019 to 31 December 2021, indicating that the nucleotide frequencies had not reached equilibrium. Figure 1 shows the bar plots of the changes in U content of the SARS-CoV-2 genomes and the sample dates. The number of Us increased in the SARS-CoV-2 genome in the same period. In the upper panels of Figure 1, it can be seen that the observed frequencies of U and C on 31 December 2021 are slightly different from the estimated trend line, but these differences are not significant (*P* > 0.05). The lower panels of Figure 1 show the changes in the number of Gs and As, respectively. The number of Gs and As were almost unchanged throughout the same time period. Solid lines in Figure 1 are trend curves of changes in nucleotide contents predicted by the new time-irreversible model. The dotted curves are the 99% confidential intervals of the predicted nucleotide contents. These curves indicate that Cs will decrease almost linearly with time, while Ts will increase all over the world.

### A global trends of nucleotide substitution rates of the SARS-Cov-2 genome

Supplementary Figures S1–S6 show bar plots of the changes in nucleotide contents of the SARS-CoV-2 genomes and the sample dates observed in Africa, Asia, Europe, North America, Oceania, and South America, respectively. Solid lines of Supplementary Figures S1–S6 also show trend curves of changes in nucleotide contents predicted by the new time-irreversible model. The same trend of nucleotide substitutions was observed in all regions.

Supplementary Figures S7–S11 show bar plots of the changes in nucleotide contents of the SARS-CoV-2 genomes and the sample dates observed in several of the dominant strains, namely, Alfa, Beta, Gamma, Delta and Omicron, respectively. Solid lines of Supplementary Figures S7–S11 are trend curves of changes in nucleotide contents predicted by the new time-irreversible model. The results indicate the trend of nucleotide changes is indeed a global pattern across all SARS-CoV-2 strains.

### Synonymous and nonsynonymous changes of SARS-Cov-2 genes

Table 4 shows the number of nonsynonymous and synonymous substitutions per site of the SARS-CoV-2 genes estimated by Nei and Gojobori model (16). In this table, $dn$ indicates the number of nonsynonymous substitutions per site and $ds$ indicates the number of synonymous substitutions per site. Except S, $dn/ds$ ratio is smaller than one. In total, $dn/ds$ ratio is 0.47.

**Table 4.** Synonymous and nonsynonymous substitutions

CDS	Start	End	d$n\ \times$1000	$ds\ \times$ 1000	$dn/ds$	Length
ORF1a	265	13468	0.57	1.36	0.42	4400
ORF1b	13467	21555	0.54	1.15	0.47	2695
S	21562	25384	1.71	1.51	1.13	1273
ORF3a	25392	26220	2.23	5.93	0.38	275
E	26244	26472	5.98	19.50	0.31	75
M	26522	27191	2.35	6.88	0.34	222
ORF6	27201	27387	13.54	54.40	0.25	61
ORF7a	27393	27759	8.74	11.05	0.79	121
ORF7b	27755	27887	10.04	38.74	0.26	43
ORF8	27893	28259	3.95	13.60	0.29	121
N	28273	29533	4.41	4.87	0.91	419
ORF10	29557	29674	11.17	43.22	0.26	38
Total			1.31	2.80	0.47	9743

## Discussion

In this study, we proposed a method for predicting nucleotide changes in SARS-CoV-2 genomes. The results shown in Figure 1 and Supplementary Figures S1–S6 demonstrate that persistent changes in nucleotide frequencies in the SARS-CoV-2 genome. In addition, comprehensive analysis presented in Figure 1 and Supplementary Figures S1–S6 showed that the high C-to-U substitution rate is not limited to any one continent but is widespread worldwide. Sequence analyses of SARS-CoV-2 revealed that the estimated nucleotide composition calculated by our method was consistent with the observed changes in nucleotide composition.

The proposed method is based on time-irreversible model described in equation. When there is a stationary distribution of nucleotide content, i.e. $\pi = \ ( {\pi ( 1 ),\ \pi ( 2 ),\ \pi ( 3 ),\ \pi ( 4 )} )$, and the detailed balance condition described in equation (3) is satisfied for all $i$ and $j$ in the stationary state, the process is time reversible (3).


(19) \begin{equation*}p\left( {t,\ i,\ j} \right)\pi \left( i \right)\ = \ p\left( {t,\ j,\ i} \right)\pi \left( j \right)\end{equation*}

These asymmetric substitutions between C and U indicate that traditional time-reversible substitution models cannot be applied to the evolution of SARS-CoV-2 sequences.

In this study, it is assumed identical substitution rates except for C-to-U in equation (3). It is possible to incorporate a more complex model. Let us assume that u and v are transition and transversion rates, respectively. The difference in rates between transitions and transversions can be taken into account by modifying equation (3) as follows:


(20) \begin{equation*}{{{\bf R}}}_1 = \begin{pmatrix} -2v-h & \quad h & \quad v & \quad v \\ u & \quad -3a & \quad v & \quad v \\ v & \quad v & \quad -3a & \quad u \\ v & \quad v & \quad u & \quad -3a \end{pmatrix} \end{equation*}

(21) \begin{equation*}{{{\boldsymbol S}}}_1 = {{{\boldsymbol Q}}}_1{{{\boldsymbol R}}}_1{{\boldsymbol Q}}_1^{ - 1} = \begin{pmatrix} 0 & \quad 0 & \quad 0 & \quad 0 \\ 0 & \quad -h-u-2v & \quad 0 & \quad 0\\ 0 & \quad 0 & \quad -2u-2v & \quad 0 \\ 0 & \quad 0 & \quad 0 & \quad -2u-2v \end{pmatrix},\nonumber\\ \end{equation*}

where


(22) \begin{equation*}{{{\bf Q}}}_1 = \begin{pmatrix} 1 & \quad -h^{2}-hu+2v^{2} & \quad 0 & \quad 1\\ 1 & \quad -hu+u_{2}-2v^{2} & \quad 0 & \quad 1\\ 1 & \quad hv-uv & \quad 1 & \quad -1 \\ 1 & \quad hv-uv & \quad -1 & \quad -1 \end{pmatrix}. \end{equation*}

Thus, we obtain substitution matrix ${{{\bf P}}}_1( t )$ by:


(23) \begin{eqnarray*} && {{{\bf P}}}_1\left( t \right) = {\mathrm{\ }}{{\bf Q}}_1^{ - 1}\nonumber\\ && \begin{pmatrix} 1 & \quad 0 & \quad 0 & \quad 0 \\ 0 & \quad {\rm exp}[-(h+u+2v)t] & \quad 0 & \quad 0\\ 0 & \quad 0 & \quad {\rm exp}[-2(v+u)t] & \quad 0 \\ 0 & \quad 0 & \quad 0 & \quad {\rm exp}[-4vt] \end{pmatrix}{{{\bf Q}}}_1\nonumber\\ \end{eqnarray*}

Equation (23) is, however, difficult to handle to estimate *h*, *u* and *v*. Previous studies showed that the rate of G-to-U is higher among transversions in SARS-CoV-2 (17,18). Further study is needed to refine the model of the evolution of SARS-CoV-2 genome.

### Nucleotide substitution rates of the SARS-Cov-2 genome

Using the new time-irreversible model of nucleotide substitutions proposed in this study, nucleotide substitution rates were estimated. The results suggest that the C-to-U substitution rate is 10 times higher than the rates of other types of substitutions. Hoshino *et al.* used the general time-reversible model with invariable sites and gamma distribution among site rate variation (GTR + G + I) as a nucleotide substitution model. The estimated mean substitution rate was $7.13 \times {10}^{ - 4}$ substitutions per site per year (95% highest posterior density interval, $6.16 \times {10}^{ - 4}-8.16 \times {10}^{ - 4}$) (19). This estimate was lower than the C-to-U substitution rates and higher than the non C-to-U of substitution rates estimated by the proposed new time-reversible model.

In this study, a simple algorithm for estimating the substitution rates using the diagonalization method is presented. The nucleotide substitution rates for the new model can be calculated as easily as with the traditional time-reversible model because the diagonalization method can be applied to the new model. To validate the new model, the number of nucleotide substitutions in genomic sequences of SARS-CoV-2 registered in the GISAID database that have been sampled from all over the world were analysed. The diagonalization method is often used for time-reversible models, such as the Hasegawa–Kishino–Yano model (9) and the general time-reversible model (11).

The changes in nucleotide contents differ among continents, as evidenced by Supplementary Figures S1–S6. However, the difference might be due to errors arising from the limited sample size, especially in Africa and Oceania. As shown in Table 1, the sample size of each continent differs substantially between continents.

### Amino acid changes and natural selection of SARS-Cov2 genes

It is widely known that mutational asymmetries affect amino acid substitutions. Jordan *et al.* found similar trends in amino acid changes across 15 taxonomic groups representing bacteria, archaea, and eukaryotes (20). Misawa *et al.* showed that these trends are mainly caused by CpG hypermutability (21). The C-to-U substitutions in SARS-CoV-2 genomes are caused by host RNA editing enzymes, such as the APOBEC family of cytidine deaminases (22–24). The C-to-U hypermutation of the SARS-CoV-2 genome will increase the number of hydrophobic amino acids in the virus proteins, because the codons of the four most hydrophobic amino acids (phenylalanine, isoleucine, leucine and valine) contain a U in the first or second position, whereas the codons of the most polar amino acids (asparagine, aspartic acid, arginine, glutamate, glutamic acid and lysine) do not contain a U in the first or second position (3,25) (see the codon table in Figure S12). The model presented in this study suggests that the number of Cs is decreasing in the SARS-CoV-2 genome, while that of Ts is increasing indicating that nucleotide frequencies have not reached equilibrium. Evolutionary studies of the SARS-CoV-2 genome must be continued to predict the future course of the COVID-19 pandemic.

Table 4 shows that the dn/ds ratio is below one, with the exception of S. In total, d*n*/d*s* ratio is 0.47. The S gene that encodes the spike protein of SARS-CoV-2, which is believed to undergo natural selection (26). As shown in Table 4, the spike protein of SARS-CoV-2, which contains 1273 amino acids, is responsible for roughly 13% of the total 9743 amino acids encoded by its genome. Hence, the predominant global trend of nucleotide variation cannot be attributed to neutral evolution (27).

### Limitations of the proposed method

It should be noted that the newly proposed method in this study may have limited applicability to RNA viruses that replicate through RNA-dependent RNA polymerases, as the C-to-U substitutions observed in SARS-CoV-2 genomes are primarily attributed to host RNA editing enzymes, such as the APOBEC family of cytidine deaminases. Additionally, in the analysis of the SARS-CoV-2 genome, the ancestral state is known. However, in cases where the ancestral state is unknown, it becomes necessary to estimate the state. Future studies are warranted to gain further insights into the evolutionary dynamics of SARS-CoV-2.

## Supplementary Material

lqae009_Supplemental_File

## Data Availability

All sequence data used in this study can be downloaded from the GISAID database (https://www.gisaid.org/). All python codes and lists of GISAID accession numbers of virus sequences used in this study are available on github (https://github.com/kazumisawa/coronavirusEvolution) and FigShare (https://doi.org/10.6084/m9.figshare.23691411). Supplementary Figures S1–S6 show bar plots of the changes in nucleotide contents of the SARS-CoV-2 genomes and the sample dates observed in Africa, Asia, Europe, North America, Oceania, and South America, respectively. Supplementary Figures S7–S11 show bar plots of the changes in nucleotide contents of the SARS-CoV-2 genomes and the sample dates observed in several of the dominant strains, namely, Alfa, Beta, Gamma, Delta, and Omicron, respectively. Figure S12 shows the standard codon table.
