# Supplementary material for: A simple method for estimating time-irreversible nucleotide substitution rates in the SARS-CoV-2 genome
Source: NAR Genom Bioinform. 2024 Feb 2;6(1):lqae009. doi: 10.1093/nargab/lqae009 (PMC11640943; doi:10.1093/nargab/lqae009)
Supplement: lqae009_Supplemental_File [file lqae009_supplemental_file.docx]

**Supplementary Materials**


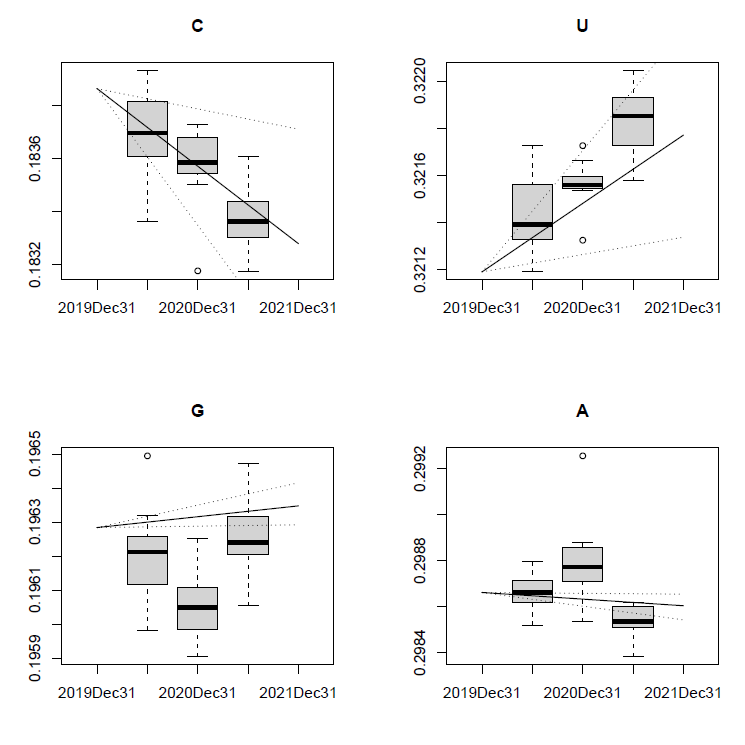


Figure S1. Bar plots of the changes in nucleotide contents of the SARS-CoV-2 genomes and sample dates observed in Africa over the time period from December 31st 2019 to December 31st 2021. Solid lines are trend curves of changes in nucleotide contents predicted by a new time-irreversible model. The dotted curves are 99 % confidential intervals of the predicted nucleotide contents.


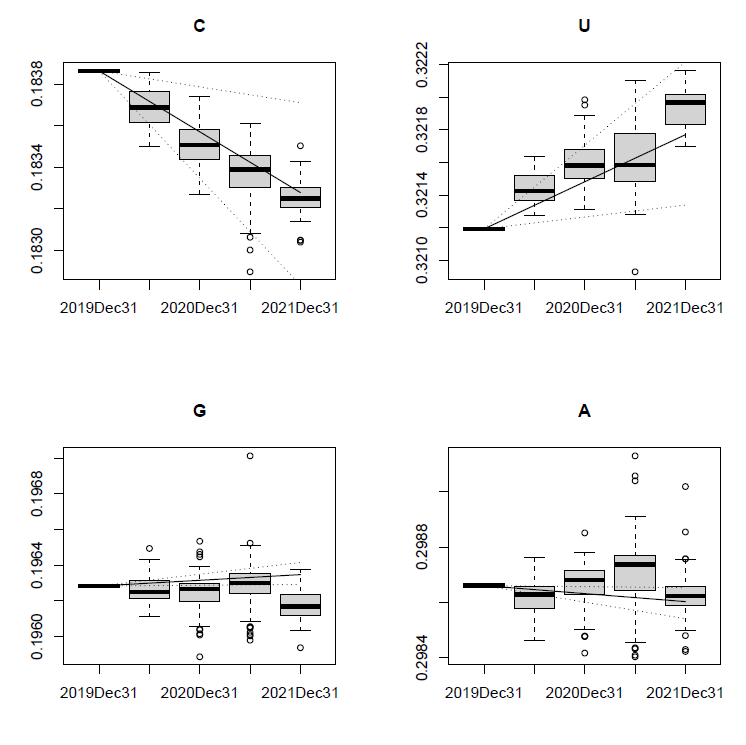


Figure S2. Bar plots of the changes in nucleotide contents of the SARS-CoV-2 genomes and sample dates observed in Asia over the time period from December 31st 2019 to December 31st 2021. Solid lines are trend curves of changes in nucleotide contents predicted by a new time-irreversible model. The dotted curves are 99 % confidential intervals of the predicted nucleotide contents.


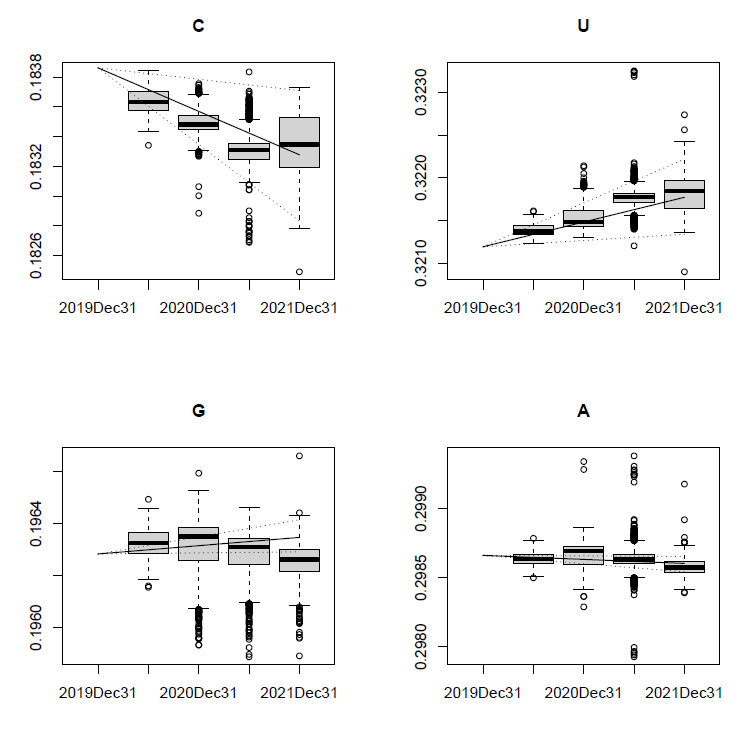


Figure S3. Bar plots of the changes in nucleotide contents of the SARS-CoV-2 genomes and sample dates observed in Europe over the time period from December 31st 2019 to December 31st 2021. Solid lines are trend curves of changes in nucleotide contents predicted by a new time-irreversible model. The dotted curves are 99 % confidential intervals of the predicted nucleotide contents.


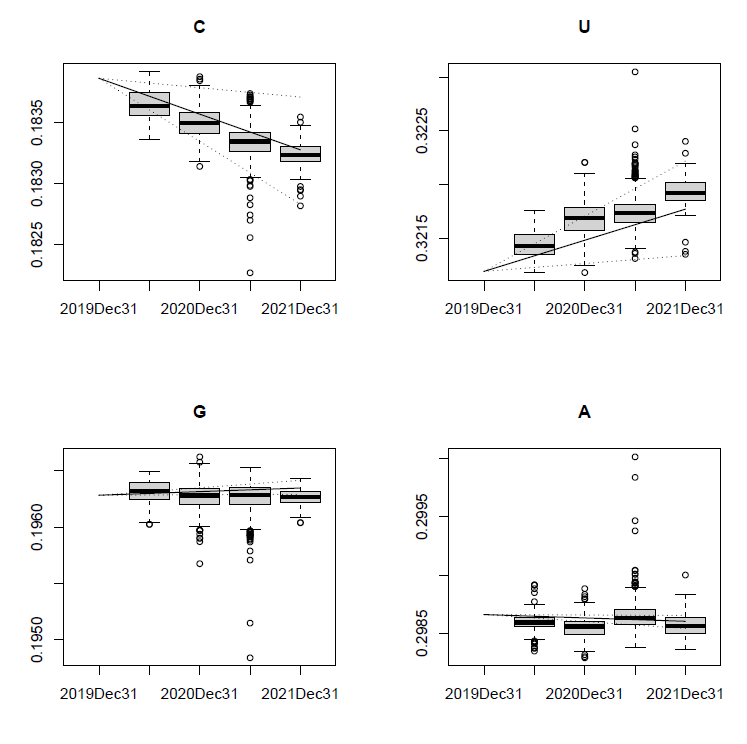


Figure S4. Bar plots of the changes in nucleotide contents of the SARS-CoV-2 genomes and sample dates observed in North America over the time period from December 31st 2019 to December 31st 2021. Solid lines are trend curves of changes in nucleotide contents predicted by a new time-irreversible model. The dotted curves are 99 % confidential intervals of the predicted nucleotide contents.


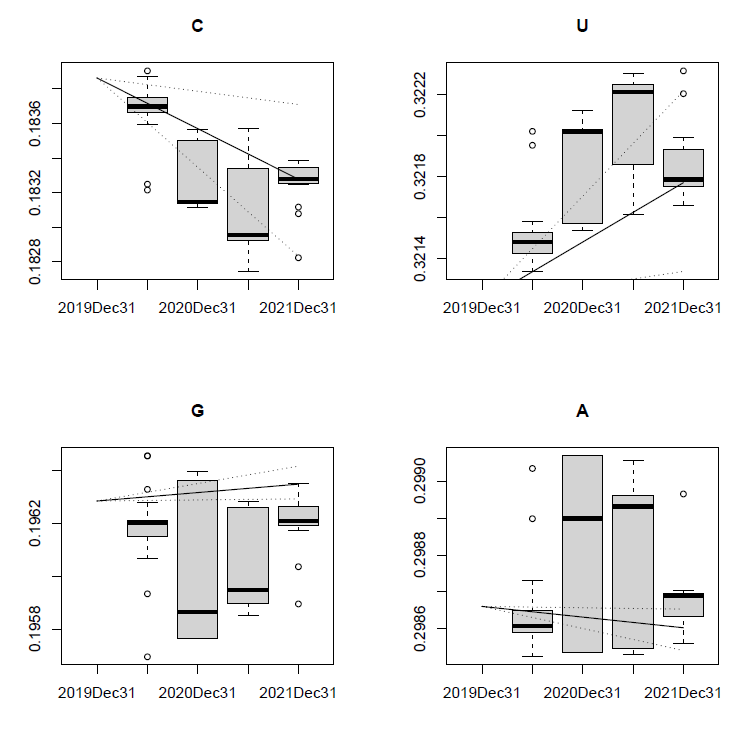


Figure S5. Bar plots of the changes in nucleotide contents of the SARS-CoV-2 genomes and sample dates observed in Oceania over the time period from December 31st 2019 to December 31st 2021. Solid lines are trend curves of changes in nucleotide contents predicted by a new time-irreversible model. The dotted curves are 99 % confidential intervals of the predicted nucleotide contents.


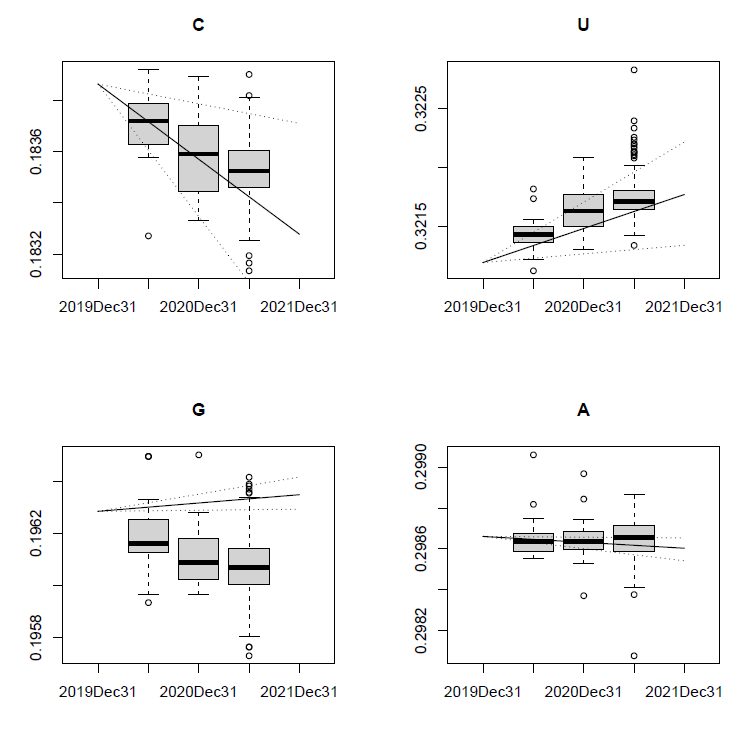


Figure S6. Bar plots of the changes in nucleotide contents of the SARS-CoV-2 genomes and sample dates observed in South America over the time period from December 31st 2019 to December 31st 2021. Solid lines are trend curves of changes in nucleotide contents predicted by a new time-irreversible model. The dotted curves are 99 % confidential intervals of the predicted nucleotide contents.

**
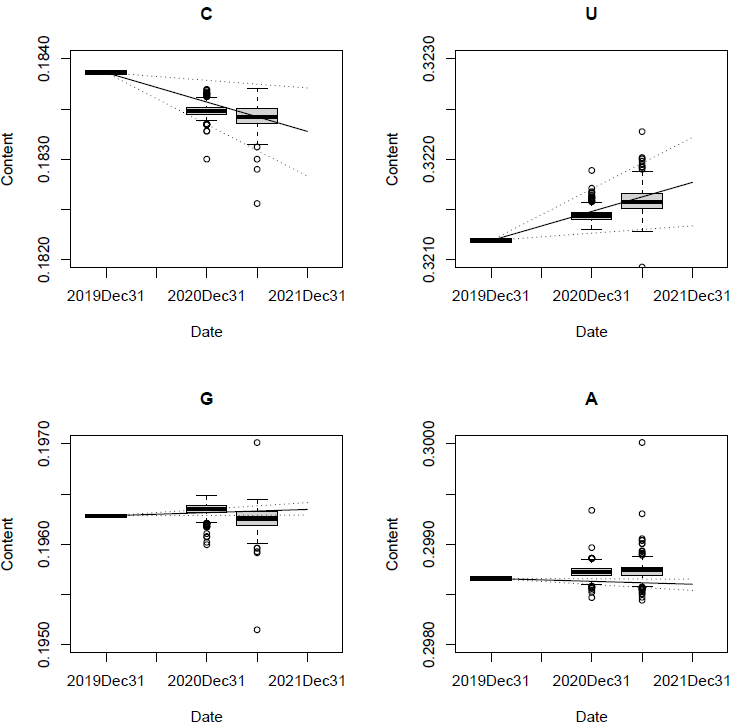
**

Figure. S7. Bar plots of the changes in nucleotide contents of the SARS-CoV-2 genomes and sample dates observed of Alpha strain over the time period from December 31st 2019 to December 31st 2021. Solid lines are trend curves of changes in nucleotide contents predicted by a new time-irreversible model. The dotted curves are 99 % confidential intervals of the predicted nucleotide contents.

**
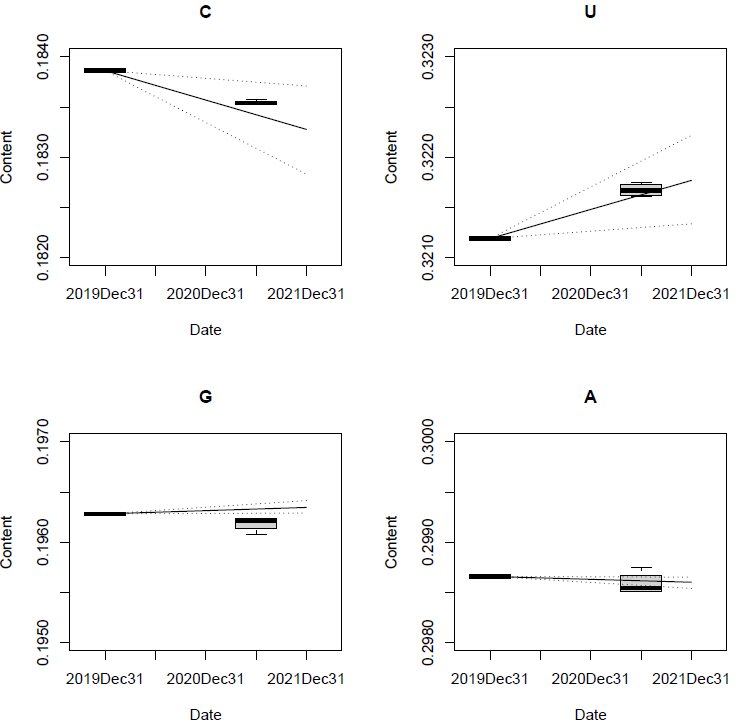
**

Figure S8. Bar plots of the changes in nucleotide contents of the SARS-CoV-2 genomes and sample dates observed of Beta strain over the time period from December 31st 2019 to December 31st 2021. Solid lines are trend curves of changes in nucleotide contents predicted by a new time-irreversible model. The dotted curves are 99 % confidential intervals of the predicted nucleotide contents.

**
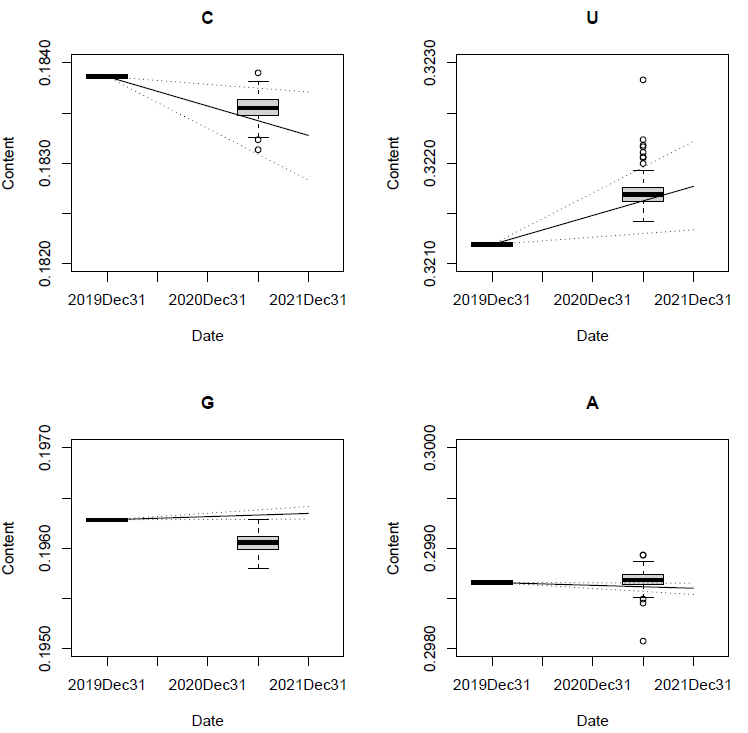
**

Figure S9. Bar plots of the changes in nucleotide contents of the SARS-CoV-2 genomes and sample dates observed of Gamma strain over the time period from December 31st 2019 to December 31st 2021. Solid lines are trend curves of changes in nucleotide contents predicted by a new time-irreversible model. The dotted curves are 99 % confidential intervals of the predicted nucleotide contents.

**
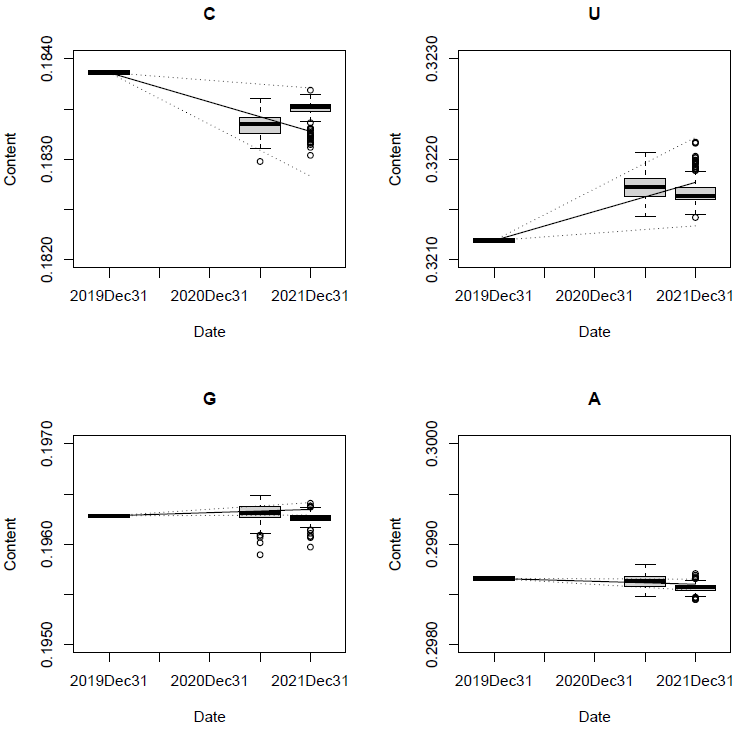
**

Figure S10. Bar plots of the changes in nucleotide contents of the SARS-CoV-2 genomes and sample dates observed of Delta strain over the time period from December 31st 2019 to December 31st 2021. Solid lines are trend curves of changes in nucleotide contents predicted by a new time-irreversible model. The dotted curves are 99 % confidential intervals of the predicted nucleotide contents.

**
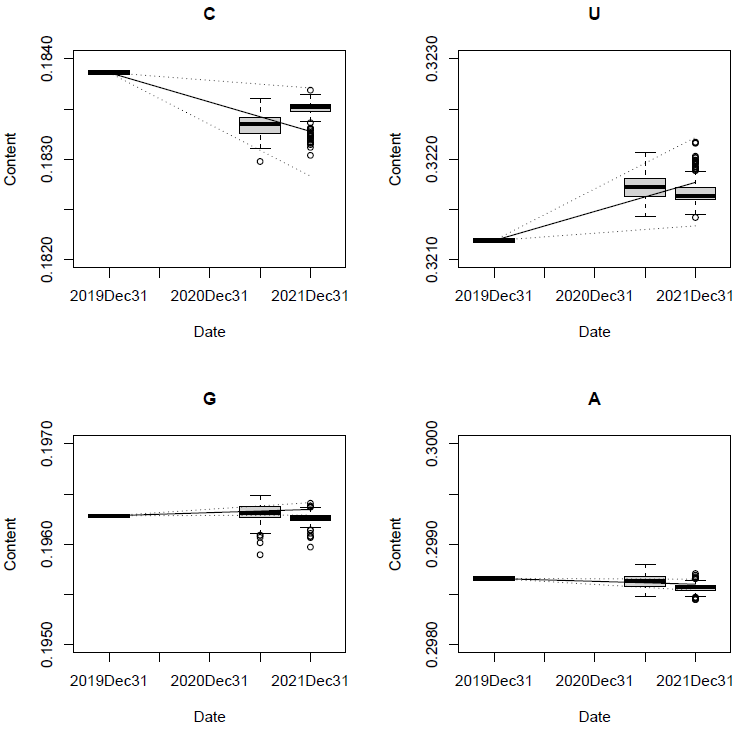
**

Figure S11. Bar plots of the changes in nucleotide contents of the SARS-CoV-2 genomes and sample dates observed of Omicron strain over the time period from December 31st 2019 to December 31st 2021. Solid lines are trend curves of changes in nucleotide contents predicted by a new time-irreversible model. The dotted curves are 99 % confidential intervals of the predicted nucleotide contents.


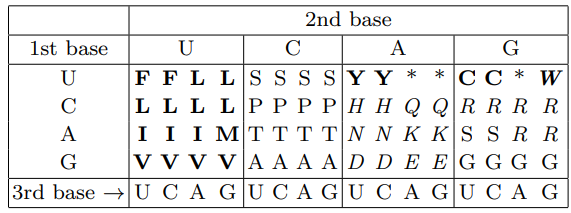


Figure S12. Codons and amino acid hydrophobicity. Bold: Hydrophobic amino acid, Italic: Hydrophilic amino acids, A: Alanine, C: Cysteine, D: Aspartic acid, E: Glutamic acid, F: Phenylalanine, G: Glycine, H: Histidine, I: Isoleucine, K: Lysine, L: Leucine, M: Methionine, N: Asparagine, P: Proline, Q: Glutamine, R: Arginine, S: Serine, T: Threonine, V: Valine, W: Tryptophan, and Y: Tyrosine.
